# Continuous recoil-driven lasing and cavity frequency pinning with laser-cooled atoms

**DOI:** 10.1038/s41567-025-02854-4

**Published:** 2025-04-11

**Authors:** Vera M. Schäfer, Zhijing Niu, Julia R. K. Cline, Dylan J. Young, Eric Yilun Song, Helmut Ritsch, James K. Thompson

**Affiliations:** 1 JILA, NIST and Department of Physics, University of Colorado, Boulder, CO USA; 2 Max-Planck-Institut für Kernphysik, Heidelberg, Germany; 3 Institut für Theoretische Physik, Universität Innsbruck, Innsbruck, Austria

**Keywords:** Atomic and molecular interactions with photons, Quantum metrology

## Abstract

Laser-cooled gases of atoms interacting with the field of an optical cavity are a versatile tool for quantum sensing and the simulation of quantum systems. These systems can exhibit phenomena such as self-organization phase transitions, lasing mechanisms, squeezed states and protection of quantum coherence. However, investigations of these phenomena typically occur in a discontinuous manner due to the need to reload atomic ensembles. Here we demonstrate hours-long continuous lasing from laser-cooled ^88^Sr atoms loaded into a ring cavity. The required inversion to produce lasing arises from inversion in the atomic-momentum degrees of freedom, which is linked to the self-organization phase transitions and collective atomic recoil lasing observed previously only in a cyclic fashion. We find that over a broad parameter range, the sensitivity of the lasing frequency to changes in cavity frequency is significantly reduced due to an atomic loss mechanism, suggesting a potential approach for mitigating low-frequency cavity noise. Our findings open opportunities for continuous cavity quantum electrodynamics experiments and robust and continuous super-radiant lasers.

## Abstract (web summary)

Experiments with cold atoms in optical cavities are often limited to discontinuous operation due to reloading requirements. Now, continuous lasing is demonstrated with strontium atoms in a ring cavity, stabilized by atom loss mechanisms.

## Main

To achieve the sufficiently cold temperatures and high phase-space densities necessary for quantum simulation^1–6^, sensing^7–12^ and lasing^13–15^, atoms are typically cooled by sequentially applying periods of different types of atomic cooling, causing the experiments to operate in a naturally pulsed fashion. Developing continuous cold atom sources will greatly advance sensing^16^, extend the length of quantum simulations and increase the quantum gate depths that can be achieved with neutral atoms. Recent advances include the continuous production of a Bose–Einstein condensate^17^, continuous transport of atoms in a lattice^18,19^, continuous loading into a high-finesse optical ring cavity^19^, and continuous replenishment of a large ytterbium tweezer array for quantum computation and simulation^20^. Lasing in cold atoms has been observed by establishing optical inversion on narrow-linewidth atomic transitions^14,15,21,22^, with the potential to realize robust, active frequency references. Lasing has also been realized using Raman transitions between atomic internal ground states^22–26^, inversion on a virtual ground state^27^ and Mollow gain on two-level optical transitions^24,28–30^. The strong coupling between atoms and the light field and their interdependency also lead to feedback mechanisms that can stabilize otherwise unstable states^31–33^.

**Fig. 1:**
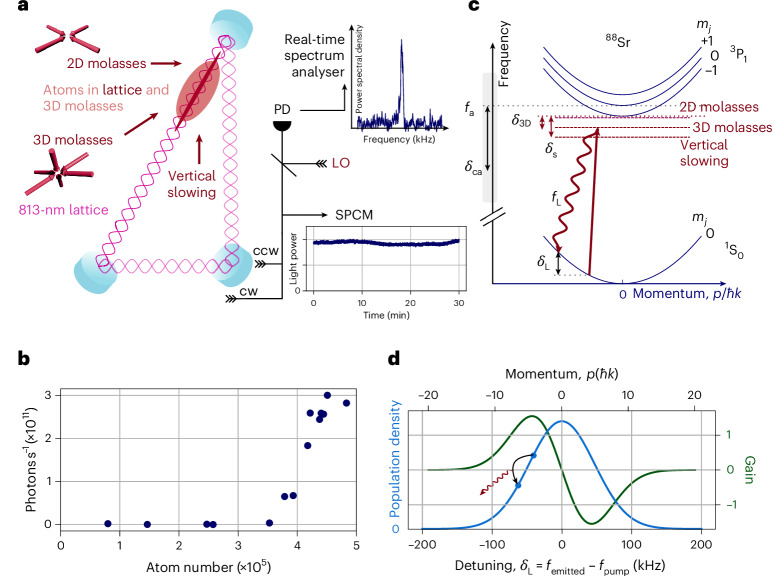
Experimental setup. **a**, Atoms are continuously loaded into a 150-μK deep 813-nm lattice inside a high-finesse ring cavity from a 3D molasses (red cloud). The lattice consists of two counterpropagating 813-nm beams (pink standing wave), and can transport atoms along the cavity axis^19^. Several cooling lasers overlap with atoms in the cavity mode: the 2D molasses, 3D molasses and vertical slowing beams. The *s*-polarized laser light exiting the cavity is coupled into a fibre and can be analysed either in the time domain on an SPCM or in the frequency domain on a real-time spectrum analyser via a heterodyne beatnote with a local oscillator (LO) beam derived from the same laser as the cooling beams. Both cw and ccw directions of the cavity are monitored. **b**, Lasing output power shows a clear threshold behaviour relative to the atom number in the cavity mode, reaching 3 × 10^11^ photons s^–1^ or approximately 90 nW at the highest atom number. Statistical errors (s.d.) are included in the size of the markers. **c**, All three cooling lasers interact with the 7.5-kHz-wide ${689}\,{\mathrm{nm}}^{1}{\mathrm{S}}_{0}^{{m}_{j} = 0}$ to ^3^P_1_ transition (*f*_a_, *m*_*j*_ = 0) with an excited-state Zeeman splitting of Δ*f* = ±1.2 MHz (Methods). The lasers are red-detuned from the closest excited state ${3\atop}{{\rm{P}}}_{1}^{{m}_{j} = -1}$ (and hence, the dominant scattering channel) by *δ*_2D_ = –80 kHz, *δ*_3D_ = –900 kHz and *δ*_s_ = –1.6 MHz. The detuning of the bare cavity resonance frequency *f*_c_ is defined with respect to the atomic resonance frequency (*f*_a_, *m*_*j*_ = 0) as *δ*_ca_ = *f*_c_ – *f*_a_. Absorption of a cooling photon at frequency *f*_3D_ and the subsequent emission of a photon at *f*_L_ with detuning *δ*_L_ = *f*_L_ – *f*_3D_ into the cavity mode changes the momentum of the atoms by ~*ℏk*, which is partly absorbed by the lattice. **d**, Momentum-state distribution of the atoms can be well approximated by the Maxwell–Boltzmann distribution, which determines the probability distribution for the frequency of the emitted photons inducing a Raman gain proportional to the gradient of the Maxwell–Boltzmann distribution. The momentum-state population difference is the maximum at one standard deviation, where one, thus, has the maximum gain as seen in RIR spectroscopy^37,56^.

We continuously load strontium atoms into a high-finesse ring cavity^19^ in which they are trapped using an 813-nm intracavity optical lattice (Fig. 1a). The laser cooling required for loading the atoms is primarily accomplished by using a continuous three-dimensional (3D) red molasses at wavelength *λ*_a_ = 689 nm. There is an additional vertically oriented slowing beam at 689 nm to facilitate capture into the 3D molasses. Once a threshold number of atoms is reached, we observe continuous light emission from the cavity, also at 689 nm, lasting for hours (Fig. 1b). The threshold atom number for lasing is *N* ≈ 300,000. At this atom number, the collective dispersive cavity shift *NU*_0_, with single-atom light shift ${U}_{0}/2\uppi =\frac{{(g/2\uppi )}^{2}{\delta }_{{\rm{ca}}}}{{\delta }_{{\rm{ca}}}^{2}+{(\gamma /2\uppi )}^{2}}=12(2)\,{\rm{Hz}}$, is much larger than the cavity linewidth *κ* = 2π × 50 kHz, leading to strong nonlinear effects and self-organization of the atoms^34,35^. Here the cavity coupling is *g* = 2π × 3.5 kHz, the atom–cavity detuning *δ*_ca_ ≈ 1 MHz and the excited-state linewidth *γ* = 2π × 7.5 kHz.

The appearance of lasing in this system is surprising at first, as lasing requires inversion between two quantum states, and there is no obvious dedicated pump mechanism applied to establish inversion. Raman lasing between Zeeman sublevels is excluded since ^88^Sr, the isotope of strontium used here, has only a single ground state (Fig. 1c). Last, Mollow gain (for example, via a three-photon process) is excluded based on the observed frequency of the emitted light^24,28–30^.

However, the molasses cooling itself creates a very cold and close-to-thermal ensemble with more atoms concentrated at lower-momentum states than higher-momentum states. Thus, there is population inversion between radial-momentum states differing by about one-photon recoil momentum. This inversion creates two-photon Raman gain between the momentum states^36^ in which a pump photon is absorbed and a lower-frequency photon is emitted, with the corresponding atom moving from a lower-momentum state to a higher-momentum state. This gain has been termed recoil-induced resonance (RIR) gain^37^ and can lead to lasing if it is sufficiently large to balance cavity losses. The predicted gain curve under simplifying approximations for the work here is shown in Fig. 1d (Methods)^37^. Only the transverse-momentum states provide gain because the axial motion of the atoms is largely frozen out since the one-dimensional optical lattice localizes each atom at a lattice antinode to much less than *λ*_a_. Pulsed lasing driven by RIR gain has been observed using a Bose–Einstein condensate and thermal atoms^38–40^. In a related regime, collective atomic recoil lasing (CARL)^36,38–41^ has been observed by pumping a ring cavity in one direction and observing the build up of backscattered light, with the underlying gain mechanism being the same as in RIR gain and with connections to free-electron lasers^42^. The collective enhancement in pump scattering can also be viewed as arising from the atoms spontaneously forming an atomic density grating from which the pump light scatters. The molasses cooling here both continuously replenishes the atoms in the cavity and repumps the atoms back to low-momentum states to continuously maintain inversion and hence gain, as was observed transiently for CARL^38,40^. Here it is additionally associated with stabilizing the lasing frequency in time compared with that without laser cooling.

Our system operates in a cross-over regime in which the cavity linewidth is comparable to or smaller than the gain linewidth^14^, limiting sufficient gain to low temperatures and a narrow spectral region. Yet, when scanning the cavity frequency, the lasing frequency does not follow it but remains constant over a large range. We identify a self-regulated atomic loss mechanism tied to cavity heating^43^ that keeps the effective dressed cavity frequency constant and close to resonant with the frequency of the the recoil-induced gain.

**Fig. 2:**
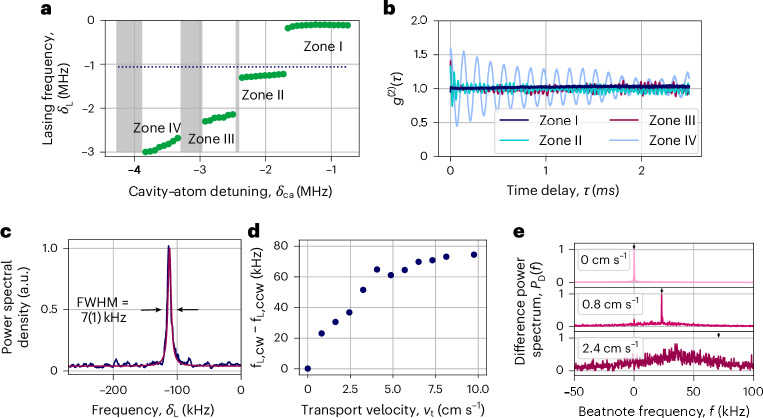
Characterization of emitted light. **a**, When scanning the bare cavity detuning *f*_c_, four zones of light emission can be identified, differing by the frequency of the emitted light *δ*_L_. Between zones II and III and zones III and IV, there are dark zones with no light emission (grey-shaded areas). The cooling lasers closest in frequency are the 3D molasses at *δ*_L_ = 0 and the slowing beam at *f*_s_, indicated by the dotted line. Errors (s.d.) are included in the size of the markers. **b**, *g*^(2)^ correlation function for the different zones. For zone I, *g*^(2)^(*τ*→0) = 1 and *g*^(2)^(*τ*) = 1 throughout, confirming coherent continuous-wave laser emission. For zones II–IV, amplitude oscillations cause oscillations in *g*^(2)^(*τ*), which become larger in amplitude and periodicity for higher-up zones. **c**, Beatnote of the lasing light in zone I with a local oscillator derived from the cooling laser has a linewidth of FWHM = 7(1) kHz. **d**, Frequency difference of the light emitted into cw (*f*_L,cw_) and ccw (*f*_L,ccw_) directions when transporting the atoms along the cavity axis at different velocities. The transport velocity is defined as *v*_t_ = *δ*_t_*λ*_813_. For up to 1 cm s^–1^, the frequency difference follows the expected Doppler shift. Above this velocity, the measured frequency difference decreases again, as the atoms are no longer pinned inside the lattice and, therefore, do not travel with the lattice velocity anymore. **e**, Beatnote between the cw and ccw emitted light has an FWHM linewidth of <18(1) Hz for a stationary lattice (Fourier limited by the measurement time) and <200 Hz for small transport velocities (*v*_t_ ≲ 0.8 cm s^–1^). For faster transport (*v*_t_ ≳ 2.4 cm s^–1^), coherence between the two lasing directions is lost. The black arrows on the top of each plot indicate Doppler-shift frequencies.

To better understand the nature of our lasing mechanism, we have characterized the frequency, frequency dependencies, coherence, linewidth, directionality and dependence on pinning of the atoms inside a lattice of the emitted light. A scan of the empty cavity mode detuning from the atomic transition frequency *δ*_ca_ = *f*_c_ – *f*_a_ over a large range, keeping all other operating conditions constant, reveals that the frequency and power of the emitted light exhibit four distinct regimes. We label these regimes as zones I–IV (Fig. 2a). Here the emitted light frequency *f*_L_ is shown via its detuning *δ*_L_ = *f*_L_ – *f*_3D_ from the frequency of the 3D molasses laser light *f*_3D_. As *f*_c_ is scanned, the lasing frequency undergoes discontinuous jumps, in some cases by more than 1 MHz.

**Fig. 3:**
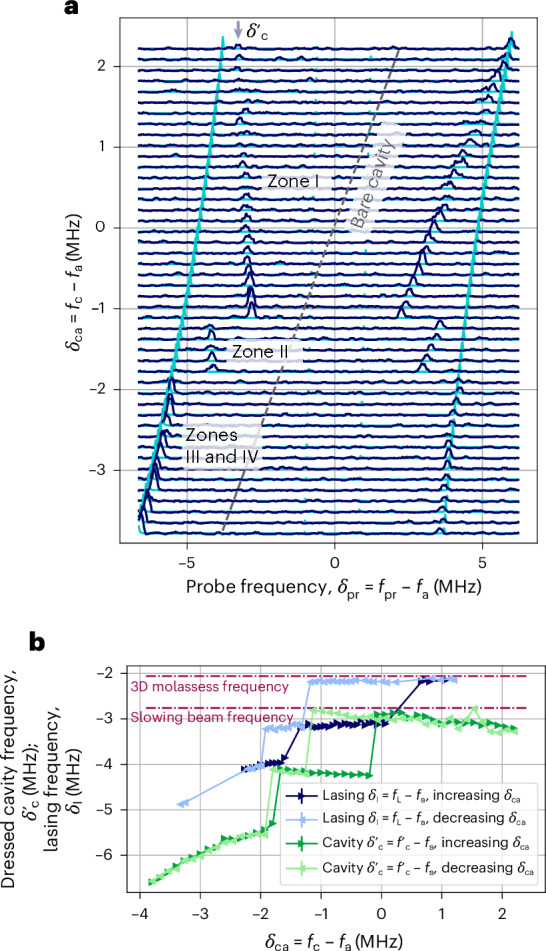
Pinning of the cavity frequency. **a**, Dressed cavity modes are observed from the cavity transmission of a weak probe beam. Lasing occurs near the dressed cavity mode ${f}_{{\rm{c}}}^{{\prime} }$ determined by fitting the transmission peak at *δ*_pr_ < 0. The observed avoided crossing (dark blue) in zones I and II does not correspond to the one expected for a constant atom number (simulation shown in turquoise). For instance, in zone I, ${f}_{{\rm{c}}}^{{\prime} }$ changes by <100 kHz over a range of > 3 MHz of *f*_c_, which would normally correspond to a change in the dressed cavity frequency of $\Delta {f}_{{\rm{c}}}^{{\prime} }=1.3\,{\rm{MHz}}$. This pinning of the cavity frequency is achieved by a self-regulated reduction in the atom number in the cavity as *f*_c_ changes. In zones III and IV, the atom number is independent of *f*_c_. **b**, Hysteresis of lasing: the frequency of the dressed cavity and emitted light for different scan directions of the bare cavity frequency *f*_c_ show a strong hysteresis with a large difference between zone transitions. When adiabatically changing the detuning *f*_c_, the transition between zones occurs later since only a small change in atom number is necessary. The dressed cavity frequency is measured only after the excited-state population has decayed, leading to a frequency offset compared with the frequency during lasing. From this offset, the excited-state population during lasing can be inferred. Errors (s.d.) are included in the size of the markers.

Each zone exhibits different pulling coefficients of the light frequency with respect to changes in the bare cavity frequency *p*_c_ = d*f*_L_/d*f*_c_. Remarkably, lasing in zone I is highly insensitive to the cavity frequency, changing by <50 kHz as the cavity frequency is changed by >3 MHz, corresponding to a cavity pulling coefficient of only *p*_c_ = 8(2) × 10^−3^ (Fig. 3a). As shown later, this insensitivity to the cavity resonance frequency emerges from a lasing-induced atom loss mechanism that stabilizes the dressed cavity resonance frequency.

We identify zone I as being associated primarily with scattering from the 3D molasses lasers. The emitted light is only ~100 kHz to the red of the 3D molasses frequency. Lasing in zone I stops immediately when the 3D molasses cooling/pumping laser is switched off, but can persist up to 700 ms after the vertical slowing beam is switched off, until the atom number has dropped below the lasing threshold. The 3D molasses pulling coefficient is *p*_3D,ss_ = d*f*_L,ss_/d*f*_3D_ = 0.9(1) in the steady state and *p*_3D,rt_ = 0.31(5) in real time, indicating that at short timescales, the 3D molasses is in stronger competition with other mechanisms to determine the lasing frequency. The real-time pulling coefficients were measured by applying sudden changes in a given laser frequency and observing the change in light frequency at short timescales of <200 μs. This differs from steady-state pulling coefficients since changes in laser frequencies also change the number of atoms at longer timescales. We identify zones III and IV as being primarily associated with the slowing beam. For instance, lasing in zone III requires the vertical slowing beam to be on, but can persist for several milliseconds without the 3D molasses cooling light. Our theoretical analysis of the lasing mechanism focuses on zone I, in which lasing is the strongest, most robust and has the most narrow linewidth.

The measured Glauber second-order correlation function *g*^(2)^(*τ*) of light is shown for each zone in Fig. 2b. In zone I, *g*^(2)^(*τ*) = 1 (${g}_{{\rm{I}}}^{(2)}(0)=1.01(6)$), consistent with coherent laser light emission. In zones II–IV, we observe oscillations in *g*^(2)^(*τ*), ${g}_{{\rm{II}}}^{(2)}(0)=1.3(1)$, ${g}_{{\rm{III}}}^{(2)}(0)=1.4(1)$ and ${g}_{{\rm{IV}}}^{(2)}(0)=1.6(1)$, indicating the light is subthermal, but exhibits super-Poissonian fluctuations in its intensity with characteristic frequencies of 7–20 kHz. These measurements indicate that the observed light emission is not simply due to incoherent single-particle scattering of light into the cavity mode.

The measured linewidth of light in zone I continuously narrows with increasing lasing intensity. Close to the jump to zone II, the light reaches full-width at half-maximum (FWHM) = 7(1) kHz (Fig. 2c). This is considerably narrower than the cavity linewidth *κ* as one expects for lasing. The linewidth is comparable to the 7.5-kHz linewidth of the 689-nm transition, though we do not assign any physical importance to this. By contrast, in zones II–IV, the linewidth FWHM ≈ 100 kHz actually exceeds the cavity linewidth. We also note that the 813-nm optical lattice is not necessary for achieving lasing, although it helps with achieving a sufficiently high atom number to reach the lasing threshold. The threshold atom number can also be reached with only atoms in the 3D molasses, and lasing in zone I has been observed with both clockwise (cw) and counterclockwise (ccw) lattice beams switched off.

We observe that light is emitted into the the ring cavity in both cw and ccw directions. The light in the two directions appears to be phase coherent with each other with zero frequency difference (see the data at *v*_t_ = 0; Fig. 2d,e) and a relative linewidth FWHM of 18(1) Hz that we attribute to the relative path length noise in the heterodyne detectors.

To further study the role of the atomic gain medium in lasing, we broke the symmetry of the coupling to the two modes by using the 813-nm lattice to continuously transport the atoms along the cavity axis at fixed velocity *v*_t_. For transport velocities of <1 cm s^–1^, the relative frequency between the cw and ccw modes corresponds to the relative Doppler shift that one would expect for a single atom moving at *v*_t_ and emitting 689-nm light into both directions at the same frequency in its reference frame. However, for *v*_t_ > 1 cm s^–1^, the relative frequency does not grow as fast as one would predict from this simple model, and the relative frequency of the two directions approaches 80 kHz, whereas the relative linewidth grows to 50 kHz; both these observations indicate that lasing in the cw and ccw modes decouple in this regime.

Having more fully characterized the nature of the observed lasing, we wish to return to the remarkable fact that lasing in zone I persists and remains at such a constant frequency even though the bare cavity frequency is changed by >3 MHz. This range is much larger than the cavity linewidth *κ*/(2π) = 50 kHz. That is, lasing occurs even though its frequency is vastly off-resonant with *f*_c_. However, the atoms in the cavity can dress the bare cavity mode, leading to an effective dressed cavity frequency ${f}_{{\rm{c}}}^{\,{\prime} }$ that can differ substantially from the bare cavity resonance *f*_c_ (ref. ^44^). Lasing then occurs into the dressed cavity mode. To investigate the dressed cavity frequency, we drive the cavity with a weak probe beam of frequency *f*_pr_. The probe beam overlaps spatially and spectrally with the emitted light, and is not visible during lasing, as the lasing intensity is much larger than the probe intensity. Therefore, we switch off all cooling lasers for 100 μs, roughly three times the excited-state lifetime, before applying the probe beam. At this point, the probe can be detected because all lasing has ceased, whereas the atom number should be unaffected as the lattice lifetime (*τ* ≈ 1 s) is much longer. If one assumes that the atom number *N* is fixed, the observed dressed cavity frequency ${f}_{{\rm{c}}}^{{\prime} }$ displays strong deviations from its expected trajectory as we vary the bare cavity frequency *f*_c_ (Fig. 3a, turquoise curve). Fluorescence imaging of the atoms in the lattice supports that the steady-state atom number *N* varies with *f*_c_. We, thus, directly fit the observed vacuum Rabi splitting to precisely determine the steady-state *N* value at each *f*_c_.

In zone I, up to 80% of the atoms are observed to be expelled from the cavity, in such a way that the dressed cavity frequency remains relatively constant over a range of > 3 MHz of *f*_c_. This drop in atom number is entirely self-regulated, and *f*_c_ is the only external parameter that is changed. As the effective cavity frequency is close to the 3D molasses light, a larger atom number will push the effective frequency from the cavity-cooling regime to the cavity-heating regime until the atom number is small enough^45^. This is closely related to heating via the RIR mechanism, as discussed in more detail in Methods. As this works only close to cavity–pump resonance, an excessively large atom number shifts the effective frequency far from the cavity–pump resonance, rendering the heating process too slow compared with the replenishing of new atoms. Hence, the position of the zone jumps is strongly hysteretic (Fig. 3b), and the cavity frequency has to be changed slowly to observe the full extent of a lasing zone. A comparison between ${f}_{{\rm{c}}}^{\,{\prime} }$ and the lasing frequency shows identical zone jumps but an offset between the lasing frequency and the dressed cavity frequency. This difference is due to the cooling lasers having been switched off for 100 μs in the measurement of the dressed cavity frequency, leading to a decay of the entire excited-state population by the time ${f}_{{\rm{c}}}^{\,{\prime} }$ is measured. The characteristic interaction strength of the atoms with the cavity that leads to the cavity mode dressing is $\propto \sqrt{({N}_{{\rm{g}}}-{N}_{{\rm{e}}})}2g$, where 2*g* is the single-particle vacuum Rabi frequency. This leads to a larger vacuum Rabi splitting at the time of the dressed cavity frequency measurement relative to when lasing is happening. From the deviation of these two frequencies, the excited-state population during lasing can be inferred. It corresponds to roughly 30% throughout. The probe transmission can also be observed in real time during lasing on a spectrum analyser by sweeping the probe frequency at a fixed rate. These measurements confirm that during lasing, the lasing and dressed cavity frequencies are identical to within ~10 kHz.

We were able to construct a phenomenological model that qualitatively captures both observed pinning of the lasing frequency in zone I and observed hysteretic behaviour of lasing when varying the bare cavity resonance frequency. The basic elements of the idea are that lasing occurs near the dressed cavity frequency, the dressed cavity frequency depends on the atom number, the atom number decreases when lasing is strong and the lasing strength decreases if the dressed cavity mode is far from resonance with the peak gain frequency. The equilibrium lasing configuration is reached when these coupled degrees of freedom are self-consistent with one another.

In more detail, RIR gain favours lasing at its maximum gain frequency, corresponding to the peak momentum-state inversion. For instance, in zone I, the measured radial atomic temperature of 10(2) μK predicts that the RIR gain is maximal at a frequency of –50 kHz to the red of the 3D molasses frequency, in rough, though imperfect, agreement with the observed –100-kHz red-detuning of the lasing light. A laser’s frequency is approximately determined by the relative linewidth of the gain medium and cavity mode, with lasing set by an appropriately defined weighted average of the cavity and gain centre frequencies^38,40,46^. In our system, the predicted RIR gain linewidth is not much narrower than the cavity linewidth; therefore, lasing can be pulled to the dressed cavity resonance frequency. When lasing, the atom number in the cavity *N* depends on the lasing strength parameterized by the average number of intracavity photons *M*. In turn, this means that the dressed cavity frequency ${f}_{{\rm{c}}}^{{\prime} }$ is tuned by the strength of lasing.

**Fig. 4:**
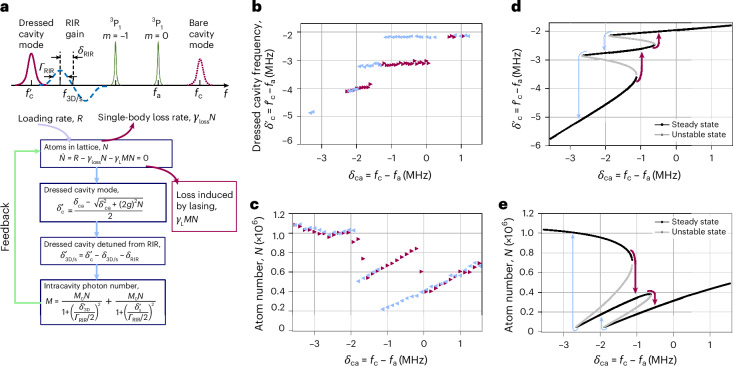
Phenomenological model. **a**, We can model the observed behaviour using rate equations. The 3D molasses gives rise to a fixed loading rate *R*, balanced by the normal atom loss from the lattice proportional to the atom number *N*, as well as an additional loss mechanism proportional to the emitted light with photon number *M*. For a fixed cavity frequency, these processes are in equilibrium, leading to a steady-state atom number *N*. The atom number affects the dressed cavity frequency, which, in turn, affects the number of photons inside the cavity through its resonance condition. Finally, the photon number is also influenced by the width and frequency of the RIR resonance with respect to the dressed cavity frequency. The final lasing frequency is a compromise between the dressed cavity frequency and the raw RIR peak. **b**–**e**, This model reproduces the emergence of different zones of stable light emission with different cavity pulling coefficients and modulation of the atom number leading to pinning of the cavity frequency, as shown by the data in **b** and **c** and the corresponding simulations in **d** and **e** (red: increasing *δ*_ca_ in time; light blue: decreasing *δ*_ca_ in time). Errors (propagated s.d.) in **b** are included in the size of the markers.

We capture this complex dynamics using the rate equation model shown in Fig. 4. In this model, all the parameters are extracted from measurements and no free-floating fit parameters were used (Methods). To capture multiple zones of lasing, we must introduce RIR gain resonances associated with both 3D molasses and slowing lasers. We numerically solve for the solutions of the coupled equations shown in Fig. 4d,e. The black curves are the solutions stable to small perturbations and the grey curves are the solutions unstable to small perturbations. The model qualitatively reproduces both observed hysteresis and frequency pinning, as well as the stepwise suppressed cavity pulling coefficients in zones II and I compared with zones III and IV. The separate zones are due to competition between the different cooling lasers, and the number of zones increases with the introduction of more cooling lasers at different frequencies.

In summary, we have observed and explained the emergence of a narrow-linewidth continuous-wave lasing mechanism that—through self-regulated feedback on the atom number—has a 120-fold suppressed sensitivity to the bare cavity frequency. We have shown how competition between different cooling lasers leads to the emergence of distinct phases of lasing with different characteristics, and how the self-organization of strongly correlated atoms can lead to the steady-state expulsion of more than 80% of the atoms from the cavity. Our lasing mechanism is coherent between both cw and ccw modes of the ring cavity and persists even when transporting atoms along the cavity axis at small enough speeds.

In conceptually related work with cold atoms, a four-wave mixing process requiring phase matching^24^ places the atoms in a superposition of momentum states, leading to spatial self-organization^34,47–52^ that can be identified with the phases of the dissipative Dicke model^24,53–55^. The self-organization can be viewed as a density grating that collectively enhances the scattering of light into the cavity that lasts for some short amount of time. In RIR such as here (and related physics of CARL), the atoms are also placed in a quantum superposition of momentum states, leading to the spontaneous formation of a moving atomic density grating that can be viewed as the mechanism for the scattering of the molasses pump light into the cavity. However, here the atoms collectively emit light into the cavity and are then reset to states of lower momentum via single-particle photon scattering during laser cooling (that is, similar to standard three- or four-level lasers). By contrast, typical self-organization physics experiments operate without entropy removal via single-particle scattering, essentially using collective four-wave mixing processes that can be mapped to a pseudo-spin Hamiltonian in certain limits.

Our experiment demonstrates how continuous cold atom experiments can lead to the emergence of new phenomena that involve hysteretic or bistable behaviour and are, therefore, difficult to reach in pulsed experiments. The self-regulated pinning of the cavity frequency over a range of several megahertz might have interesting future applications in metrology. In the future, we will exploit the continuous nature of the loading and laser cooling to realize narrow-linewidth continuous super-radiant lasing on the 1.3-mHz transition in ^87^Sr, with important applications for metrology and quantum sensing. The work here inspires the consideration of new approaches to generate self-regulated pinning of the cavity mode for realizing additional insensitivity to cavity frequency noise beyond the previously identified approach of working in the deep bad cavity limit.

## Methods

### Loading and transport

^88^Sr atoms are continuously loaded into a lattice inside a ring cavity of finesse ${\mathcal{F}}=33,000$ (at 689 nm, *s* polarization), single-atom cooperativity *C* = 0.16 and cavity coupling *g* = 3.5(2) kHz. Up to 1.1 × 10^6^ atoms are loaded in the steady state, and cooled to a temperature of 10 μK. For loading, the atoms are heated in an oven, slowed in a Zeeman slower and then gradually cooled via a vertical chain consisting of a blue (461-nm) two-dimensional (2D) magneto-optical trap, a red (689-nm) 2D magneto-optical trap, a 689-nm 2D molasses and finally a 689-nm 3D molasses overlapping spatially with the lattice^19^. In addition, a vertical slowing beam (also 689 nm) reduces the vertical velocity of the atoms. The two magneto-optical trap stages are separated from the lattice region by a baffle with a small hole for the atoms, but light from the 2D molasses, 3D molasses and vertical slowing beam spatially overlap with the atoms in the lattice. The three cooling lasers have respective detunings of *δ*_2D_ = –80 kHz, *δ*_3D_ = –900 kHz and *δ*_s_ = –1.6 MHz from the ${1\atop}{\rm{S}}_{0}^{{m}_{j} = 0}$ to ${3\atop}{{\rm{P}}}_{1}^{{m}_{j} = -1}$ transition. Both 2D molasses beams are retro-reflected and propagate parallel to the table, with each beam at an angle of ~45° with respect to the plane of the cavity. They are located above the cavity, and only the lowest ~10% of the beams overlaps with atoms interacting with the cavity mode. The three roughly orthogonal 3D molasses beams are also retro-reflected and span all three dimensions of space, where all the beams were designed to have an ~45° angle to the table as well as the plane of the cavity. This corresponds to angles of 69°, 69° and 43° with respect to the cavity axis, which is oriented at 20° from the vertical direction. Due to spatial constraints, the actual beam geometry deviates slightly from the symmetric arrangement, and the angles of the three beams with respect to the cavity axis are 75°, 65° and 55°. The slowing beam is almost orthogonal to the table and only propagates upwards. The cavity axis into which the atoms are loaded has a 70° angle with respect to the table. The cavity has a linewidth of *κ*/2π = 50 kHz at 689 nm and is locked to the 689-nm laser. The quantization axis is defined by a magnetic field 30° out of plane relative to the cavity axis, leading to mixed polarizations for all cooling lasers. This magnetic field leads to a Zeeman splitting of Δ*f* = ±1.2 MHz of the excited states in ^3^P_1_. The cavity also supports a lattice produced by two counterpropagating 813-nm beams, which are locked in frequency to the ring cavity. The lattice has a depth of 150 μK, corresponding to an axial frequency of 210 kHz and a radial frequency of 440 Hz. The lattice differential light shifts and the Zeeman shifts cause the relevant optical atomic transitions at 689 nm to shift to the red by –1.3 MHz, –0.7 MHz and 1.8 MHz for *m*_*j*_ = –1, 0 and 1, respectively, relative to the transition frequency *f*_a_ at zero magnetic field and no 813-nm lattice. A travelling lattice to transport the atoms along the cavity axis is produced by introducing a frequency difference between the two lattice beams.

### Homodyne analysis

Both cw and ccw output modes of the ring cavity are fibre coupled, filtered for 689-nm light and analysed on a single-photon-counting module (SPCM). Typical count rates on the SPCM during lasing are 1.5 MHz. For the *g*^(2)^ measurement, the SPCM output is recorded and time-tagged on an analogue-to-digital converter and the *g*^(2)^ correlation function is estimated via

1 $${g}^{\,(2)}(\tau )=\frac{\mathop{\sum }\nolimits_{i = 1}^{{i}_{\max }-\tau }{n}_{i}{n}_{i+\tau }}{{\left(\mathop{\sum }\nolimits_{i = 1}^{{i}_{\max }-\tau }{n}_{i}\right)}^{2}}\left({i}_{\max }-\tau \right),$$

where *n*_*i*_ are the counts detected in bin *i*, *τ* is the time delay from the beginning of the measurement and *i*_max_ is the total number of bins. The dead time of the SPCM is 22 ns, and the bin time was chosen to be 300 ns. For coherent light, *g*^(2)^(0) = 1, whereas for the Fock states, *g*^(2)^(0) = 0; for thermal light, *g*^(2)^(0) = 2. Since we are using a single SPCM and no beamsplitter, the dead time of the SPCM means that coincidence counts arriving within the dead time are not detected. For excessively short bin times, this artificially reduces *g*^(2)^(0) below 1. Due to the cavity ring down time of *t*_rd_ = 3.2 μs, most coincidence counts are nevertheless detected in this setup without the need for a beamsplitter and two SPCMs. For excessively large bin times, the influence of other correlations, such as intensity fluctuations, on *g*^(2)^(0) increases. The 300-ns bin time was chosen as a compromise between the two effects.

For measuring the dressed cavity frequency, a weak probe beam is inserted into the cavity. Since the probe beam overlaps spatially and spectrally with the lasing light, it is not visible during lasing. To measure ${f}_{{\rm{c}}}^{\,{\prime} }$ during lasing, all cooling lasers are switched off for 100 μs, during which the excited state decays and lasing ceases. After 100 μs, the probe beam is switched on and its transmission is measured for a single probe frequency *f*_pr_ and cavity frequency *f*_c_. The cooling lasers are then switched on again to reload atoms and the measurement is repeated for a different *f*_pr_ value, leading to one horizontal trace for a fixed *f*_c_ value. Finally, the cavity frequency is changed and the measurement is repeated.

### Real-time heterodyne analysis

For analysing the frequency of the emitted light, the fibre-coupled cavity output is overlapped on a non-polarizing beamsplitter with a local oscillator (~1-mW power) derived from the same laser as the cooling lasers. The frequency of the local oscillator is shifted such that its beatnote with the lasing light is at around 5–8 MHz. Both outputs of the non-polarizing beamsplitter are guided to the two ports of a differential photodiode, and the relative intensities are carefully matched using a glass pick-off to achieve the maximum cancellation of common-mode noise. The differential photodiode signal is then amplified by 50 dB, leading to a signal up to ~15 dB above the noise floor at the peak lasing intensity. The beatnote is analysed on a real-time spectrum analyser, and can be time resolved to about 30 μs. This setup can also be used to measure ${f}_{{\rm{c}}}^{{\prime} }$ during lasing, as the lasing light has a narrower linewidth than the cavity. For this purpose, the probe beam is swept in frequency over a range of >100 kHz at a rate of about 1 Hz during lasing. The dressed cavity frequency is then visible as a periodically appearing broader resonance on top of the continuous signal of the lasing beatnote. This measurement also shows the real-time shifting of the dressed cavity frequency when switching off the cooling lasers.

### RIR

RIR provides a gain mechanism in cold atom systems by the population inversion of momentum states^37^. In ring cavities, this can lead to a collective instability—CARL—via coherent collective backscattering reminiscent of free-electron lasing^38^. The interference between the pump and backscattered fields mediated by the momentum states lead to a spatial atomic density modulation transverse to the cavity axis, which then enhances backscattering and collectively accelerates the atoms. Adding extra friction can lead to a quasi-continuous backward coherent emission. In our system, pumping is provided by the cooling lasers, which are scattered into the cavity mode. For modelling this, we look at the radial momentum of the atom cloud, as the atoms are tightly confined in the Lamb–Dicke regime in the axial direction. In the radial direction, the confinement is weaker, and the atoms can move within each pancake orthogonal to the cavity axis experiencing the polarization and intensity gradient from the molasses beams. Each scattering process is accompanied by a recoil transfer depending on the angle of pump and cavity photon momenta during absorption/emission. The axial part of the momentum will be absorbed by the lattice so that only the radial projection is important. Although molasses cooling generates a more complex distribution, for simplicity, we assume a thermal distribution of the momentum states of the atoms along the radial direction as described by the Maxwell–Boltzmann distribution:

2 $${\rho }_{{\rm{p}}}(\,p)=\frac{1}{\sqrt{2\uppi m{k}_{{\rm{B}}}T}}{{\rm{e}}}^{-{p}^{2}/2m{k}_{{\rm{B}}}T},$$

where *m* and *p* denote the mass and momentum of a ^88^Sr atom, respectively; *k*_B_ is the Boltzmann constant; and *T* is the radial temperature of the atom cloud. The population inversion between two different momentum states can then be expressed as Δ*ρ*_p_(*p*, *δp*) = *ρ*_p_(*p* + *δp*) – *ρ*_p_(*p*), where the momentum transfer *δp* = *nℏk* depends on the number and direction of the photons absorbed and emitted in one recoil process. The RIR gain is proportional to the size of this population difference or inversion. The frequency at which this inversion contributes RIR gain is given by the change in kinetic energy during this momentum transfer process from *p*_*i*_ to *p*_*f*_, that is, $\Delta f=({p}_{f}^{2}-{p}_{i}^{2})/2mh$, which, for a single-photon transfer *p*_*f*_ = *p*_*i*_ + *ℏk*, amounts to Δ*f*_1_ ≈ 2*ℏkp*_*i*_/2*mh* = 2*n*_*i*_*f*_*r*_, with *p*_*i*_ = *n*_*i*_*ℏk*.

In our setup, there are three approximately retro-reflected pump lasers covering all three spatial dimensions with different angles to the lattice direction. We can therefore approximate that, on average, the momentum transfer between the absorbed pump photon and emitted photon in the radial direction equals one photon momentum *n* = 1. The light emitted by the atoms during RIR is shifted in frequency corresponding to the momentum gain of the atoms. Converting the gain from momentum into frequency units yields

3 $$\begin{array}{l}{\rho }_{f}\,(\Delta f\,)=\sqrt{\frac{m}{2\uppi {k}_{{\rm{B}}}T}}\frac{\lambda }{n}\\ \left[{{\rm{e}}}^{-{\left(\frac{2\uppi m\Delta f}{nk}+\frac{n\hslash k}{2}\right)}^{2}/2m{k}_{{\rm{B}}}T}-{{\rm{e}}}^{-{\left(\frac{2\uppi m\Delta f}{nk}-\frac{n\hslash k}{2}\right)}^{2}/2m{k}_{{\rm{B}}}T}\right]\end{array},$$

where *k* = 2π/*λ* is the wavevector of the emitted photon orthogonal to the cavity axis and Δ*f* is the frequency difference of the emitted light from the absorbed (cooling) light. For an atom cloud of *T* = 10 μK, the maximum gain, therefore, occurs at Δ*f* = 50 kHz.

### Temperature of atoms

The temperature of atoms trapped in the lattice is measured via fluorescence imaging of the thermally expanding cloud, yielding 10(2) μK. As the fluorescence of the atoms in the 3D molasses is more intense than that of atoms in the lattice, first, the 3D molasses cooling lasers are switched off for 70 ms to allow for the atoms to gravitationally fall out of the imaged area, whereas only atoms trapped in the lattice remain. 70 ms after this, the lattice beams are switched off, and the atomic cloud is imaged after different expansion delays. Since the RIR mechanism transfers atoms to a higher-momentum state, the lasing-induced loss mechanism would most intuitively stem from heating of the atoms. However, no change in the temperature of atoms is observed as the lasing intensity increases. This could be explained if the atoms are not in a thermal equilibrium state. Because the RIR gain maximum for hotter atoms is closer to the lasing frequency, the lasing-induced heating mechanism is the most efficient for already hotter atoms, which can get heated out of the lattice in a runaway manner before the cloud can thermalize. At the same time, colder atoms get cooled more efficiently due to more favourable Doppler shifts. The hotter atoms would then no longer be confined in the lattice by the time the temperature can be measured. Although this theory qualitatively explains our observations, we do not have any experimental evidence for it and can, therefore, not be certain what the nature of the lasing-induced loss mechanism is. Density-dependent mechanisms such as collisions or formation of molecules have been excluded experimentally, as no dependence of the atom loss on atom density has been observed.

### Pulsed lasing

**Extended Data Fig. 1:**
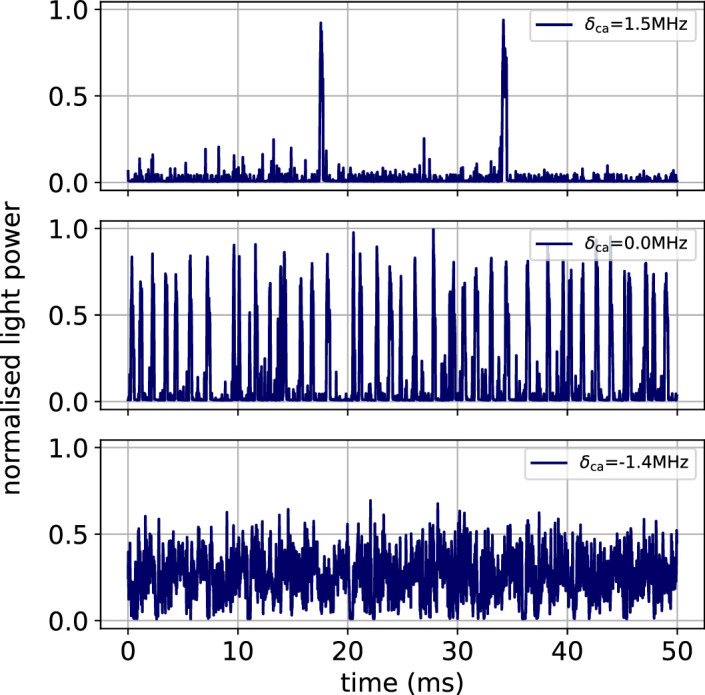
Pulsed lasing. The lasing is not continuous throughout zone I, but for positive *δ*_ca_ only occasional pulses occur. In this limit cavity cooling and self-ordering should be present, which work towards increasing the effective cavity-atom detuning, increasing the nonlinear dynamics. This can also create excess heating during the ordering process reducing the effective gain [57]. For smaller *δ*_ca_, where more atoms have be ejected from the cavity to keep the dressed cavity resonant with the lasing light the pulses are more frequent. For even smaller *δ*_ca_ the lasing is continuous.

Lasing in zone I is not continuous throughout, but operates in a pulsed regime depending on the cavity detuning *δ*_ca_ (Extended Data Fig. 1). The measured linewidth in the pulsed regime is broadened compared with the cw lasing linewidth. Measurements of the linewidth, pulling coefficients and so on were usually taken at *δ*_ca_ ≈ –1.3 MHz, close to the transition between zones I and II, where lasing is the most intense and narrow linewidth. The exact position of the jump between zones depends on the reloading rate of atoms into the lattice, which depends on the number of atoms in the 3D molasses and, therefore, the individual laser frequencies and intensities.

### Phenomenological model

We consider three different atomic loading and loss mechanisms in our rate equation model: ^88^Sr atoms are constantly filling up the one-dimensional lattice at a loading rate of *R* = 2 × 10^7^ atoms s^–1^, which is measured from vacuum Rabi splitting when turning on the lattice over an existing equilibrium 3D molasses. Atoms are lost via single-body loss characterized by the rate *γ*_loss_ = 19 s^–1^, which is calculated from the loading rate *R* and the steady-state atom number *N* = 1.1 × 10^6^ without lasing. The steady-state atom number *N* is also extracted from vacuum Rabi splitting. The second loss mechanism *γ*_L_*MN* is associated with recoil lasing. During lasing, the steady-state atom number will be the solution of the rate equation

4 $$\dot{N}=R-{\gamma }_{{\rm{loss}}}N-{\gamma }_{{\rm{L}}}MN=0.$$

The reference intracavity photon number (*M*_0_ = 1,045 photons per atom) is measured from the SPCM count rate at the edge of zones I and II in which lasing is maximal in power. The lasing-induced loss rate *γ*_L_ = 8.93 × 10^−6^ per photon per atom can be calculated from equation (4). The number of emitted photons during lasing depends on the absorption of pumping light; therefore, the intracavity photon number *M* is strongly affected by the dressed cavity frequency ${f}_{{\rm{c}}}^{{\prime} }$ and the detuning of the pumping light (${\delta }_{3{\rm{D}}}^{{\prime} }$ and ${\delta }_{{\mathrm{s}}}^{{\prime} }$) from the RIR gain:

5 $$M = \frac{M_{0} N}{1+\left(\frac{\delta_{3{\rm{D}}}^{\prime}}{{\varGamma}_{{\mathrm{RIR}}}/2}\right)^2} + \frac{M_{0} N}{1+\left(\frac{\delta_{{\mathrm{s}}}^{\prime}}{{\varGamma}_{{\mathrm{RIR}}}/2}\right)^2},$$

where

6 $$\delta_{{{ 3{\rm{D}}/{\rm{s}} }}}^{\prime} = f_{{\mathrm{a}}} + \frac{\delta_{{\mathrm{ca}}} - \sqrt{\delta\,_{{\mathrm{ca}}}^2 + (2g)^2 N}}{2} - (\,f_{{{ 3{\rm{D}}/{\rm{s}} }}} + \delta_{{\mathrm{RIR}}}).$$

Here RIR gain FWHM *Γ*_RIR_ = 50 kHz and shift *δ*_RIR_ = 100 kHz are calculated using equation (3). The intracavity photon number *M*, in turn, influences the steady-state atom number *N*, establishing a negative feedback loop.

The qualitative agreement between the equilibrium solutions of this system and our experimental observations is evident. There are overlapping regions of stable solutions, connected by unstable solutions, which cause the observed strong hysteresis. In particular, the cavity pulling coefficient is suppressed for zones I and II, but recovers for zones III and IV in which no notable atom number modulation occurs that would impact the dressed cavity resonance. The model also reproduces the substantial reduction in the atom number within zones I and II, a critical aspect for the maintenance of lasing and the pinning of the cavity resonance frequency. The emergence of these distinct zones can be attributed to the competitive dynamics between different cooling lasers. The introduction of additional cooling lasers at varying frequencies leads to a proportional increase in the number of observed and calculated zones.

## Online content

Any methods, additional references, Nature Portfolio reporting summaries, source data, extended data, supplementary information, acknowledgements, peer review information; details of author contributions and competing interests; and statements of data and code availability are available at 10.1038/s41567-025-02854-4.

## Data Availability

The data that support the plots within this paper and other findings of this study are publicly available at https://scholar.colorado.edu/concern/datasets/0v838219g.
